# Selective surface modification of lithographic silicon oxide nanostructures by organofunctional silanes

**DOI:** 10.3762/bjnano.4.22

**Published:** 2013-03-25

**Authors:** Thomas Baumgärtel, Christian von Borczyskowski, Harald Graaf

**Affiliations:** 1 Center for Nanostructured Materials and Analytics, Institute of Physics, Chemnitz University of Technology Reichenhainer Str. 70, 09126 Chemnitz, Germany

**Keywords:** AFM lithography, amino-functionalization, local anodic oxidation, octadecyl-trichlorosilane, silicon oxide nanostructures

## Abstract

This study investigates the controlled chemical functionalization of silicon oxide nanostructures prepared by AFM-anodization lithography of alkyl-terminated silicon. Different conditions for the growth of covalently bound mono-, multi- or submonolayers of distinctively functional silane molecules on nanostructures have been identified by AFM-height investigations. Routes for the preparation of methyl- or amino-terminated structures or silicon surfaces are presented and discussed. The formation of silane monolayers on nanoscopic silicon oxide nanostructures was found to be much more sensitive towards ambient humidity than, e.g., the silanization of larger OH-terminated silica surfaces. Amino-functionalized nanostructures have been successfully modified by the covalent binding of functional fluorescein dye molecules. Upon excitation, the dye-functionalized structures show only weak fluorescence, which may be an indication of a relatively low surface coverage of the dye molecules on length scale that is not accessible by standard AFM measurements.

## Introduction

Local anodic oxidation (LAO) nanolithography is a reliable and convenient method for the structuring of silicon on a nanometer scale [1–3]. The generated silicon oxide nanostructures are characterized by a height of only a few nanometers and a width down to some 10 nm [4]. After the local nanostructuring of the substrate, desired materials have to be selectively immobilized on the structures in order to provide them with an adjustable functionality. A combination of LAO with a treatment of the substrate surface by organic self-assembled monolayers (SAM) is a promising approach for a versatile combined top-down/bottom-up process towards the fabrication of functional nanodevices. Depending on the structure type, a multitude of routes have been proven applicable in a substantial number of publications [2,5–9].

In general, the different binding mechanisms that can be used for the selective functionalization of local anodic oxidation patterns on monolayer-terminated silicon can be divided into three major groups: (A) intermolecular interactions [10–11], (B) electrostatic/ionic interactions [12–13], and (C) covalent bonds [14–17]. Covalent attachment of molecules is thereby a most favorable method since covalent bonds possess comparable high dissociation energy (several 100 kJ/mol), and with the knowledge of their successful implementation on nanostructures, the whole realm of modular chemistry can be applied (e.g., “Click”-chemistry [18]). Mechanisms such as electrostatic binding or binding by intermolecular forces (e.g., polar interactions or van-der-Waals forces) on the other hand are characterized by weaker binding strengths and a lower selectivity, and thus, are not as suitable for multistep surface functionalization as covalent binding.

Although the covalent functionalization of LAO patterns has been reported several times recently, most cases involve either a local mild oxidation of the monolayer only or an etching of the silicon oxide after LAO lithography [19]. Such realizations, however, require a very fine adjustment of the oxidation parameters or an additional chemical treatment. In this study, we report on a direct covalent binding to nanolithographic silicon oxide structures. A direct binding of the functional material to the oxide nanostructure is possible if the material possesses an appropriate anchoring group that binds selectively to silicon oxide surfaces. A standard substance for chemical modification of silicon oxide surfaces are silane compounds such as trichlorosilanes or ethoxysilanes [20–21]. In order to provide the possibility for further chemical modification, functional silanes are of major interest. These molecules possess a functional head-group (e.g., carboxyl, amino or thiol group) in addition to the silane tail group. This functional head group can react with other molecules resulting in an immobilization of the desired material on the structure with the silane molecule as linker. The binding of diverse silanes to silicon oxide surfaces has been the subject of several investigations [22–24]. Though a previous study demonstrated the general feasibility to bind silane molecules to LAO nanostructures, there was strong evidence for only a partial coverage of the structure and no complete monolayer formation [25].

**Figure 1:**
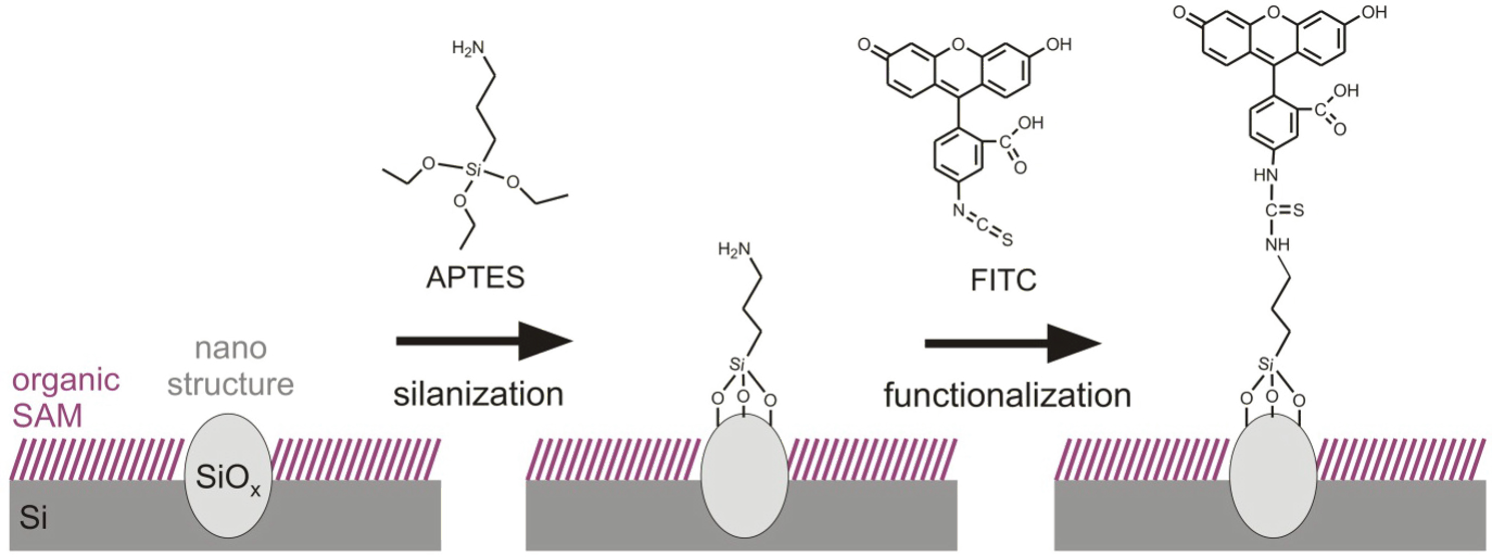
Schematic route for covalent binding of FITC to a silicon oxide nanostructure on alkyl-terminated silicon. After local anodic oxidation, a layer of APTES molecules is bound to the silicon oxide as linker. In a second step, FITC is bound to the amino-functionalized oxide structure.

Here we demonstrate a successful route for a homogeneous and dense binding of functional silane molecules to silicon oxide nanostructures prepared by LAO lithography of alkyl-terminated silicon [26]. In addition, the dye fluorescein-5-isothiocyanate (FITC) was subsequently bound to the amino-terminated silane layer. FITC is a fluorescein derivative with an N=C=S functional group. This group is reactive towards nucleophiles such as amine or thiol groups. A successful large-scale binding of FITC to amino-terminated silicon surfaces [27] has been demonstrated previously by fluorescence measurements [28]. The attachment of FITC on the oxide nanostructures is realized in two steps, as depicted schematically in Figure 1. First, the functional silane aminopropyltriethoxysilane (APTES) is bound to the LAO oxide, which leads to an amino-functionalization of the structure. In a second step, FITC is bound to the amino group on the structure through its N=C=S group. The successful binding is confirmed by AFM topography measurements and spectrally resolved fluorescence microscopy. Prior to the two-step functionalization with APTES and FITC, the quality of the silane layer formation is tested and proven by the binding of long-chained silanes, which are known to form densely packed and well-ordered monolayers on silicon surfaces [29–30].

## Results and Discussion

### Successful routes towards silane-functionalization of silicon oxide nanostructures

**Figure 2:**
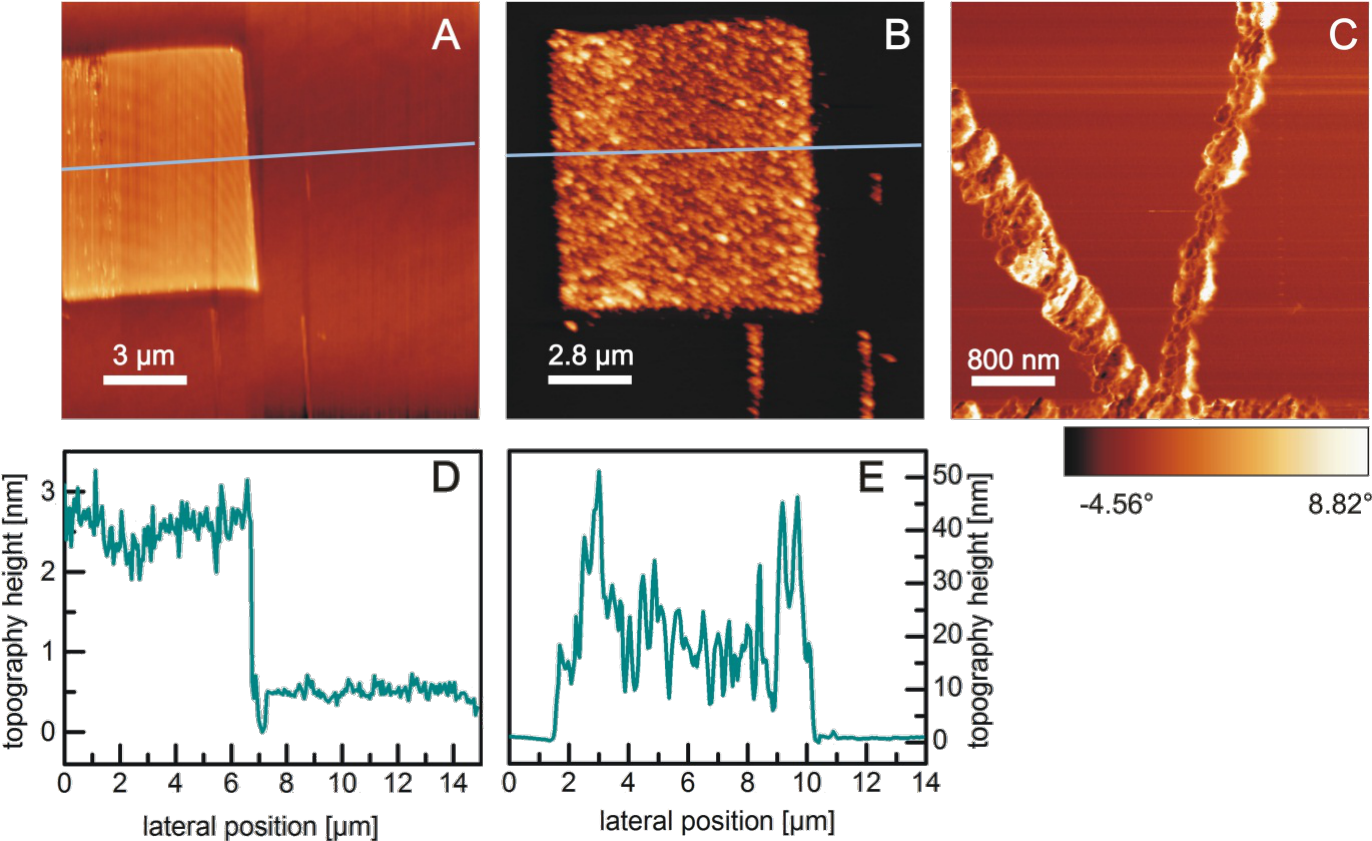
AFM height images of LAO oxide structures before (A) and after (B) 40 h in a 10 mM OTS solution in toluene under ambient conditions. (C) AFM phase image of line structures after 40 h in OTS. (D, E) topography profiles along the lines in A and B.

**Figure 3:**
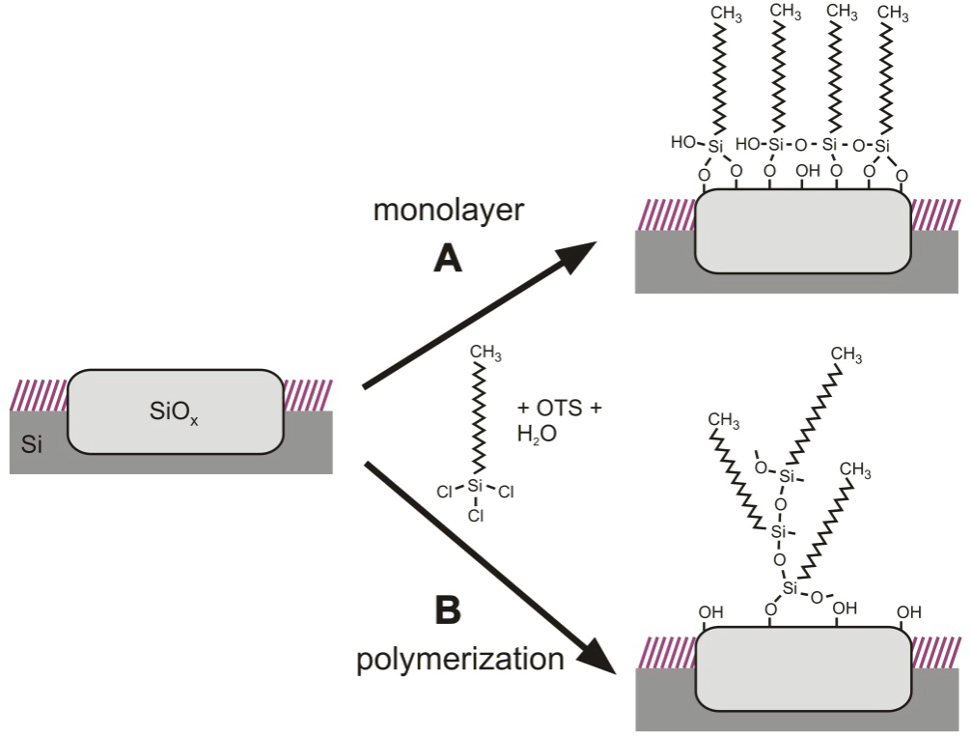
Schematic illustration of the two different possible covalent binding mechanisms of OTS with a silicon oxide surface. (A) formation of a smooth OTS monolayer, (B) vertical polymerization.

Prior to the multistep attachment of FITC through APTES linkers, the controlled and dense binding of silane molecules to the silicon oxide nanostructures has been investigated in detail. OTS (octadecyltrichlorosilane) has been used for these studies as its large molecular length allows for a reliable detection by AFM measurements, and the resulting layer thickness and smoothness is an indicator of the packing density of the molecules [23,31]. Figure 2 shows the AFM images of a square oxide structure before (A) and after (B) 40 h in a 10 mM OTS solution in toluene under ambient conditions. After the OTS treatment, the measured structure height increases from about 2 nm up to 50 nm (Figure 2D and Figure 2E). Additionally this increase is very nonuniform: there is a formation of clusters of different height (Figure 2E) and the RMS roughness on the structure increases more than one order of magnitude (0.5 to 10). The lateral structure of the formed OTS layer can be observed in Figure 2C. There is a grainy substructure, which is in the range of some 10 to 100 nm, and therefore within a similar length scale as the measured topography height variations (Figure 2E). Therefore, it can be concluded that the OTS clusters possess a globular structure. As the cluster sizes are much larger than the chain length of an OTS molecule (2.6 nm), OTS evidently does not form closed monolayers on the nanostructure (Figure 3A), but rather a vertical or 3D polymerization of OTS has to be assumed (Figure 3B). For much shorter immersion times in the OTS solution (several minutes to a few hours), only a partial and also very nonuniform height increase of the LAO structures is observed. Obviously, without any special preparation conditions, the tendency towards a 3D polymerization is much stronger than that for the formation of smooth uniform layers.

A vertical polymerization is known for tri- and di-functional silanes, especially alkoxysilanes and alkylchlorosilanes [23,32–33] under certain conditions. In order to understand why 3D polymerization is preferred to monolayer formation on the LAO structure under ambient conditions, the reaction mechanism of trichlorosilanes with silica surfaces has to be considered in greater detail.

In the presence of water, the Si–Cl bonds of an OTS molecule undergo hydrolysis forming Si–OH groups. If the OTS molecule is close to the substrate, these Si–OH groups react with OH groups on the silicon oxide surface forming a Si–O–Si bond and water [20]. Such a reaction is of course also possible between two OTS molecules leading to a cross-linking of the layer molecules. Thus, the presence of a surface water layer on the silicon oxide is a necessary condition for the monolayer formation, but on the other hand too much water on the surface also promotes vertical polymerization. As the silicon oxide nanostructure is much more hydrophilic than the surrounding alkyl monolayer, the wetting of the structure is strongly favored. Recent investigations revealed that there is a pronounced water layer formation on top of the oxide structure due to ambient humidity, which takes place on the time scale of several hundred minutes [34].

Besides the availability of water, the surface density of OH groups (silanole groups) on the silica surface has a very strong impact on whether there is a monolayer formation or a vertical polymerization [23]. If the density of such groups is sufficiently large, the hydrolyzed OTS molecules should tend to react preferentially with the surface due to steric reasons. A 3D polymerization requires a certain twisting of the molecules out of their equilibrium conformation. In order to obtain high quality monolayers, silica substrates are usually cleaned in an oxidizing acid (e.g., “piranha” solution) to provide a maximum coverage of the surface with OH groups. However, such a method is not possible for LAO nanostructures on alkyl-terminated silicon as the protecting monolayer would also be oxidized. The strong tendency towards vertical polymerization in the presence of ambient water however, is indirect evidence for a rather low density of OH groups on the lithographic oxide. This is further supported by the fact that for shorter immersion times of the substrate within the OTS solution, also a very nonuniform increase in height with clear local differences was observed. The few surface hydroxy groups also lead to a much slower binding rate of OTS to the LAO oxide. Even after several hours in the OTS solution, the surface was not fully covered with OTS, whereas for “piranha”-cleaned silica a fractional surface coverage near unity was reported within a few minutes [29,35].

**Figure 4:**
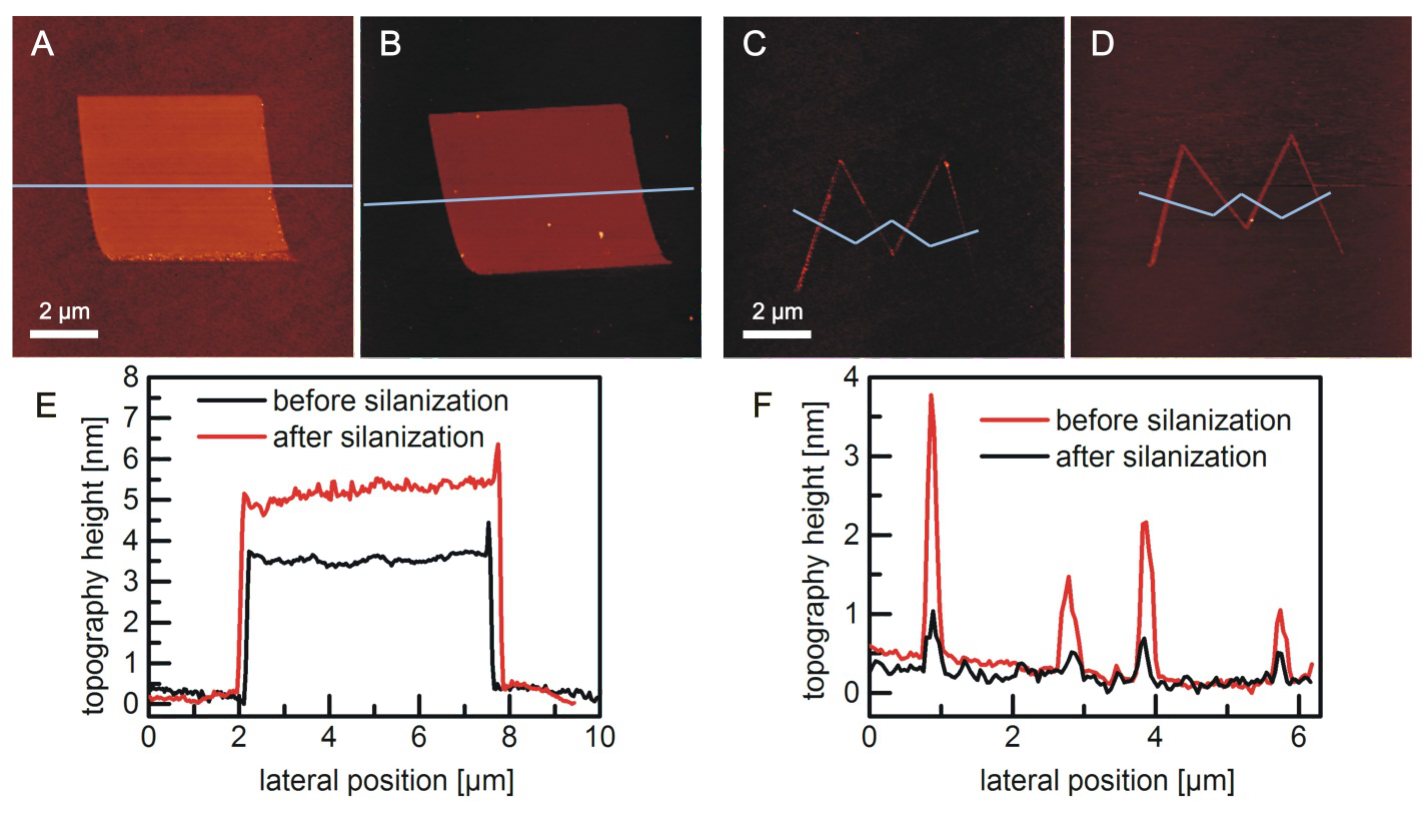
AFM height images of LAO oxide structures before (A, C) and after (B, D) 16 h in a 10 mM 11-bromoundecyltrichlorosilane solution in dry toluene under dry conditions. (E, F) topography profile along the lines in (A–D) averaged over a width of 10 pixels.

As the OH group density on the LAO oxide cannot be altered, the only way to prevent vertical polymerization is to completely remove the water from the silane solution in toluene. This was achieved by placing the vessel in a desiccator during reaction and further drying of the solvent (sparging of the solution with an inert gas for one hour before the reaction). Under such conditions, a much more uniform coverage of the silicon oxide nanostructures could be achieved (Figure 4). The small amount of water that is necessary for the hydrolysis of the trichlorosilane may originate from residual water in the dried solution or a very thin water film that is adsorbed on the oxide surface during transfer under ambient conditions. Since the rest of the alkyl-terminated silicon is much more hydrophobic than the local oxide, surface water will condense preferentially on the nanostructures.

Compared to the binding under ambient conditions, the height increase is much more uniform under dry conditions. In the case of a square structure, the formed silane layer (here: 11-bromoundecyltrichlorosilane) possesses a thickness of about (1.6 ± 0.1) to (2 ± 0.1) nm (Figure 4A, B and E). This is in good agreement with the thickness of 11-bromoundecyltrichlorosilane monolayers on silica substrates which was determined to a value of 1.81 nm by ellipsometry measurements [30]. There are also a few larger elevations, which may be due to polymerization already within the solution. Such impurities could be avoided by using better anhydrous conditions and controlled process conditions; however, they are not crucial for the general observations and their physicochemical interpretation. The height increase of the line structures (Figure 4C, D and F) is also within a reasonable range of (0.5 ± 0.1) to (2.5 ± 0.1) nm. Silane layer thicknesses that are smaller than the molecular length may be explained by a low surface coverage. If the monolayer is not densely packed, the molecules will not stand nearly upright (in all-trans conformation), but the alkyl chains will orient more parallel to the surface, which leads to a decrease of the measured layer thickness [29]. A height increase of more than the thickness of a densely packed monolayer, on the other hand, is a strong indication for the beginning vertical polymerization. It is striking that there seems to be a correlation between initial structure size and the thickness of the silane layer: the higher and wider the initial oxide, the larger is also the height increase by the binding of silane molecules. Whether this is caused by either an influence of the surface geometry on the reaction mechanism/the molecular order or possibly an artefact of the AFM measurement (water adsorption or the energy dissipation of the AFM tip) should be investigated in detail in further statistical studies.

### Covalent binding of FITC to silicon oxide nanostructures

**Figure 5:**
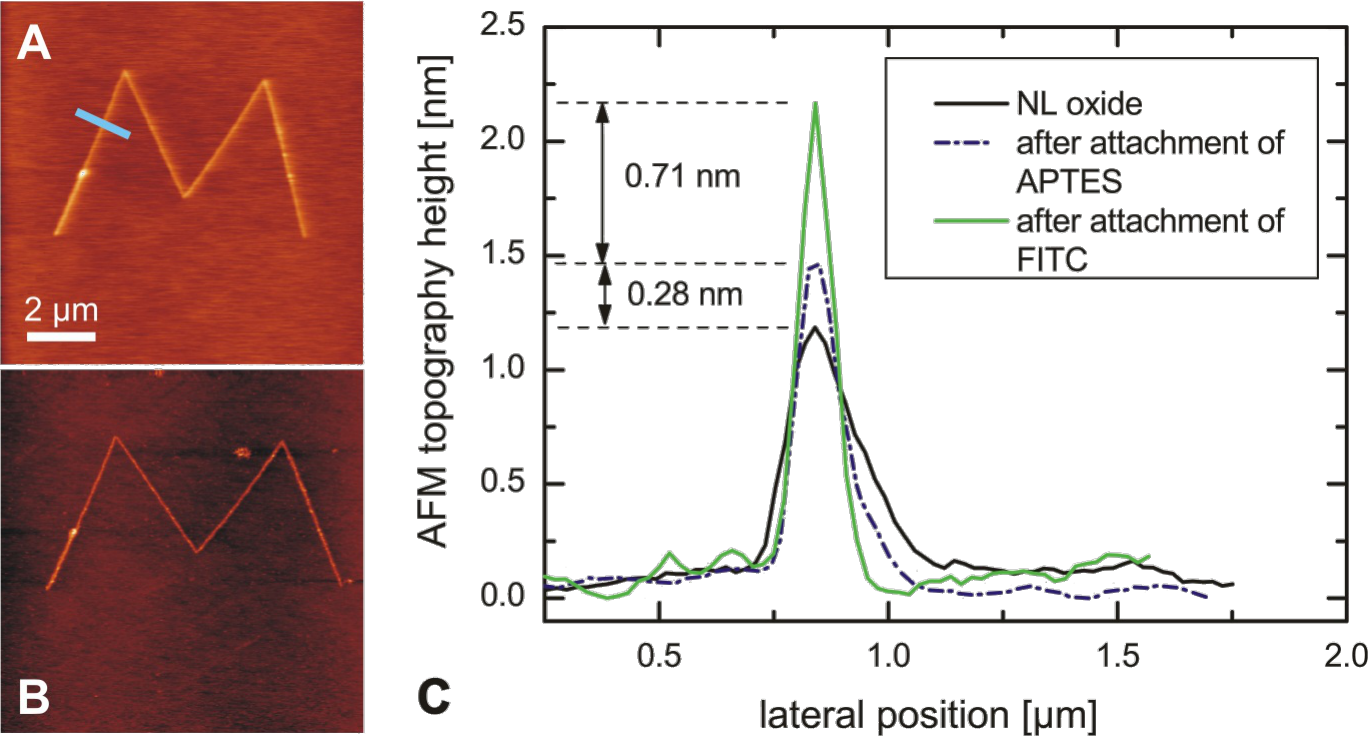
(A) and (B) AFM height images of LAO oxide line structures before any functionalization (A) and after binding of FITC (B). (C) Height profile of an LAO oxide structure (black curve) after 6 h in 10 mM APTES solution in dry toluene under dry conditions (blue dashed curve) and after subsequent binding of FITC in aqueous solution (green curve). The profiles were measured and averaged over a width of 20 pixels along the path indicated by the blue line in Figure (A).

Once suitable conditions for the controlled silanization of LAO nanostructures had been found, functionalization with fluorescent FITC molecules was carried out according to the route displayed in Figure 1. Figure 5 shows the results of comparative AFM height measurements after different functionalization steps.

After binding of APTES, there is a height increase of approximately 0.3 ± 0.1 nm (blue dashed curve), which is in good agreement with the length of a hydrolyzed APTES molecule (0.3 nm). The subsequent binding of FITC to the amino-functionalized nanostructure leads to a further increase in height of about 0.7 ± 0.1 nm. As the length of a single FITC molecule is about 1 nm, it can be concluded that the molecules are not standing upright but are tilted from the surface normal. The tilt is most likely caused by the thiocarbonyl group (planar trigonal geometry due to binding sp^2^ orbitals). A calculation of the tilt from the height measurements is not straightforward though, as too many assumptions about the 3D orientation and bending of the bound FITC molecules would have to be made. Further theoretical considerations (e.g., molecular dynamics simulations) could be employed in order to investigate the molecular orientation on the surface, but it has to be kept in mind that AFM measurements of such systems may also be influenced by many other factors, such as tip–sample interactions and the formation of water layers, that strongly depend on the chemical nature of the surface.

**Figure 6:**
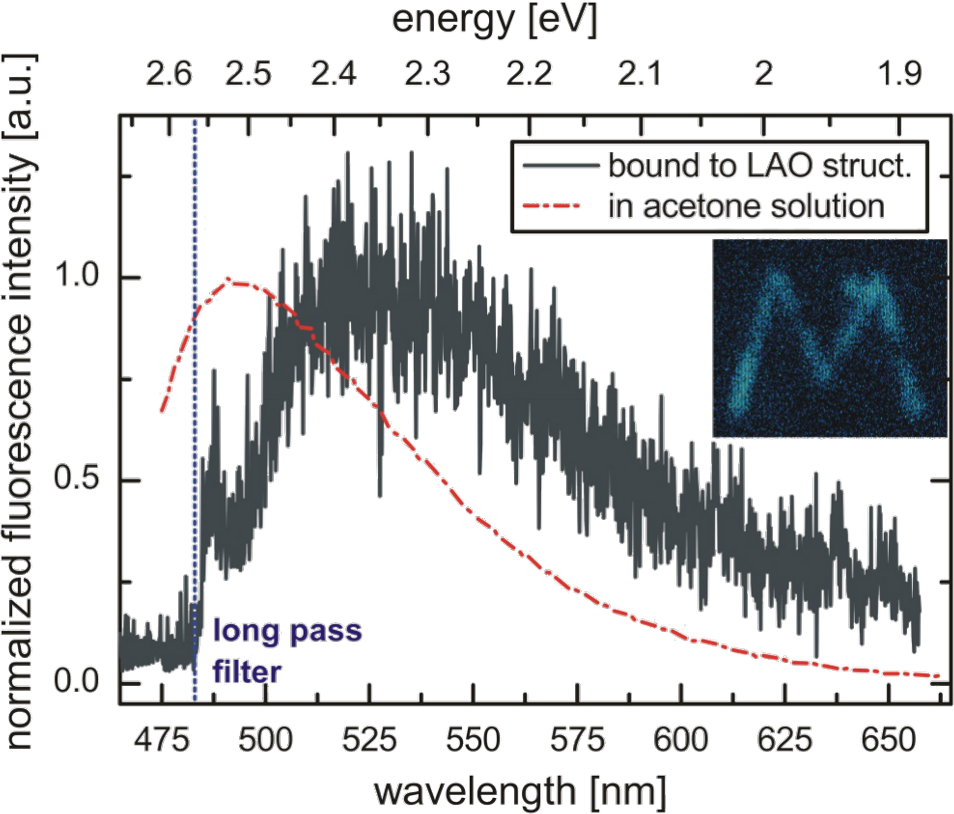
Fluorescence spectrum from the FITC-functionalized LAO oxide nanostructure shown in Figure 5 (dark grey curve) compared to FITC fluorescence in acetone solution (red dashed curve). The inset shows a confocal microscope image of the structure (excitation wavelength: 465 nm, excitation power: 11 μW, integration time: 1 ms/px).

FITC functionalized nanostructures have been investigated using fluorescence microscopy, which further confirms the successful binding. Figure 6 shows a typical fluorescence spectrum from the FITC-terminated structure depicted in Figure 5. A confocal microscope image of the whole structure is displayed in the inset. There is a clear luminescence signal, which originates from the nanostructure only. The spectral shape of the fluorescence light resembles that of FITC in acetone solution and is shifted about 36 nm to the red due to the different dielectric environment (the small signal close to the filter edge at 485 nm is not part of the FITC fluorescence but probably scattered light from the environment).

**Figure 7:**
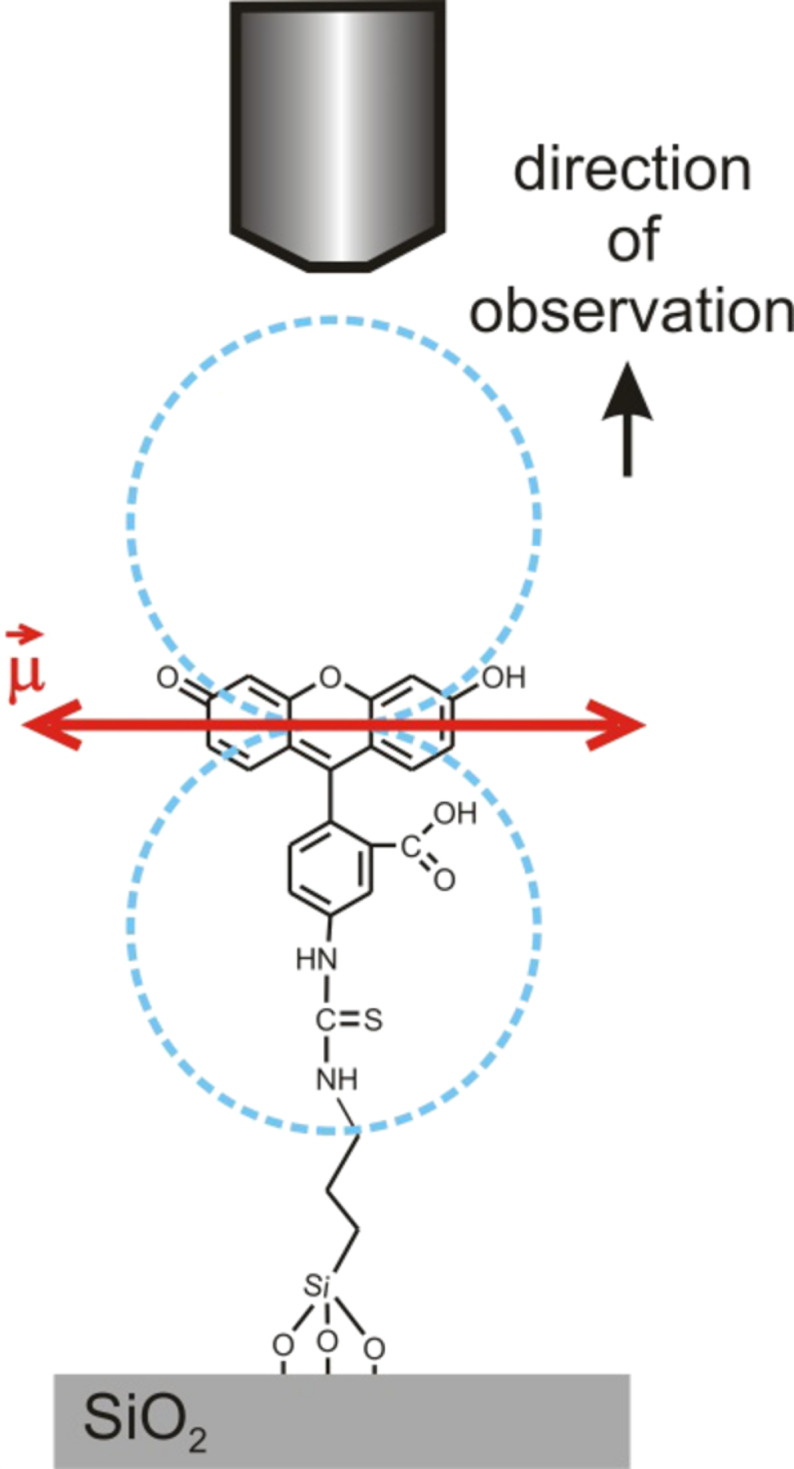
Schematic of the transition dipole moment orientation 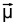 (red arrow) of FITC bound to a SiO_2_ surface.

The fluorescence signal is comparably weak (roughly a factor of 10 above the background noise level). The main reason for this is the quenching by the underlying silicon. Nevertheless, other high-quantum-yield xanthene dyes (e.g., rhodamine 6G) that are bound to the nanostructures by electrostatic interactions, show a much higher signal-to-noise ratio for similar structure heights and excitation powers [13]. Thus, the quenching by the underlying silicon does not explain the measured low fluorescence intensities. Another important factor that influences the absorption and emission of radiation by the bound molecules is the orientation of their transition dipole moment. For xanthene derivatives, such as fluorescein or rhodamine, the transition dipole moment 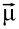 for the S_1_ → S_0_ transition is typically oriented along the xanthene unit [36–37]. As this unit is perpendicular to the binding axis of the FITC molecule, it should be oriented rather parallel to the surface (tilt angle α = 0°, Figure 7). Since the directional characteristic of a dipole scales with cos^2^α, a very large tilting (α close to 90°) would be necessary to explain the much lower fluorescence intensities. Such a large tilt is very unlikely due to steric reasons as well as the molecular structure and would also be contradictory to the AFM-investigations.

It may also be possible that the fluorescence is quenched by an efficient energy transfer between densely packed FITC molecules on the structure. However, such an effect can be excluded by comparison with the study of Imhof et al. [38]. They investigated FITC bound to silica spheres and found a quenching of the fluorescence intensity for increasing dye concentration, which was attributed to an interaction between neighboring molecules. This interaction also causes a lowering of the excited state energy, which was observed as a 10 nm red shift of the emission spectrum from 531 nm to 541 nm for increasing dye concentration. Although it is difficult to exactly determine the peak position in Figure 6 due to the low signal-to-noise ratio of the spectrum, the maximum position clearly seems to be rather in the range of 530 nm than 540 nm. The absence of the red-shift as well as the agreement of the approximate peak position and spectral shape with the results of Imhof et al. for low dye concentrations, leads to the conclusion that the observed low fluorescence intensity is likely not caused by a dense packing of the FITC molecules on the oxide structure. Measurements of the excited state lifetime are likely not suitable for the investigation of the intermolecular interactions due to the predominant quenching by the silicon below. Consequently, the most likely reason for the strong difference in fluorescence intensity between the electrostatically bound dyes and the covalently bound FITC is a much lower surface density in the case of covalently bound FITC.

Of course this conclusion, though plausible, is based on many uncertainties and assumptions. Yet a more precise quantification is very difficult due to the unknown quenching by the underlying silicon and the unavailability of suitable measurement techniques of the surface coverage for such small geometries. If the density of FITC molecules on the structures is really much lower than that of electrostatically attracted dyes then this may be a hint that either the APTES monolayer is not closed on a nanoscopic scale or the molecules in the monolayer are not well-ordered. The latter would lead to a rather low density of surface amino groups [39] that are available for reaction with the functional group of FITC. However, the next-neighbor distance between two adjacent surface amino groups must be below the AFM tip diameter (≈30 nm) as no distinct height steps or islands could be observed in the AFM images. A resolution of the density of bound molecules may be possible by using novel techniques such as the measurement of AFM amplitude–phase–distance curves [40]. From such experiments, the dissipated energy of the AFM tip oscillation can be calculated, which depends on the local elastic and therefore structural surface properties of the substrate. The surface coverage of the relatively rigid silicon oxide with “softer” organic molecules should in principle be distinguishable by the amplitude–phase–distance curve technique.

## Conclusion

In conclusion, a route for a controlled covalent functionalization of silicon oxide nanostructures with an amino-terminated silane and FITC dye molecules has been successfully realized. The formation of silane monolayers on nanoscopic silicon oxide nanostructures has proven to be much more sensitive towards ambient humidity than, e.g., silanization of larger OH-terminated silica surfaces. This is most likely due to a lower density of surface hydroxy groups, which requires a longer reaction time for the formation of a closed monolayer. Optical investigations of the bound FITC dye, on the other hand, seem to indicate that the forming APTES monolayer is not well-ordered on a scale below 20 nm. Pasternack et al. found that the ordering of APTES monolayers can be improved through in situ heating during the functionalization reaction [39]. An elevation of the reaction temperature seems to be a promising step for future experiments in order to obtain more densely packed FITC monolayers on the silicon oxide nanostructures. Also a formation of silane monolayers from the gas phase should be considered, as this technique enables a controlled formation of highly ordered films under appropriate conditions [41–43].

## Experimental

The local anodic oxidation experiments were carried out on 1 × 1 cm^2^ pieces of weakly doped n-type silicon (resistivity: >3000 Ω·cm, Fraunhofer ENAS, Chemnitz, Germany) substrates with a self-assembled monolayer (SAM) of 1-dodecene molecules as a surface coating.

The monolayer preparation was carried out in three steps: cleaning, native-oxide removal, and monolayer formation (radical chain reaction); see also [25,44]. The substrates have been cleaned by ultrasonication in acetone (C_3_H_6_O, “Uvasol^©^ for spectroscopy”, Merck, Germany), ethanol (C_2_H_5_OH, “Uvasol^©^ for spectroscopy”, Merck, Germany) and in “piranha”-solution [40% hydrogen peroxide (H_2_O_2_, 30% “Suprapur”, Merck, Germany) and 60% sulfuric acid (H_2_SO_4_, 96% “Suprapur”, Merck, Germany)] at 70 °C. Afterwards the samples were rinsed thoroughly with Millipore™ (ultraclean demineralized and deionized water, resistivity of > 18 MΩ·cm) and dried in a nitrogen stream. In the next step the silicon oxide samples were etched in aqueous hydrofluoric acid (HF, 40% “Suprapur”, Merck, Germany) solution (3–4% in volume) for 3 min at room temperature in order to remove the native silicon oxide. Subsequently, organo-silicon monolayers were prepared according to [45] by using 1-dodecene (C_12_H_25_, for synthesis, Merck, Germany) in pure form. The alkene was deoxygenated with argon gas at least 30 min prior to the reaction and the argon flow was also maintained during the reaction. After transferring the etched substrates to the 1-dodecene solution, it was heated up to 190 °C for at least 7 h. Afterwards the samples were rinsed and sonicated for 5 min in dichloromethane (CH_2_Cl_2_, “Uvasol^©^ for spectroscopy”, Merck, Germany) and ethanol followed by drying in a nitrogen stream.

LAO experiments as well as topography measurements were conducted with an Anfatec Level AFM (Anfatec Instruments AG, Germany) by using platinum-coated silicon tips (”NSC18/Pt”, resonance frequency 75 Hz, spring constant 3.5 N/m, Mikromash, Estonia). As the usage of conductive ink or tape should be omitted in order to prevent contamination during the following wet-chemical steps, the substrates were fixed on a metal sample holder with a small magnet (thus providing a back-side contact to the substrates, which is sufficient for LAO). The sample holder was electrically contacted to a voltage source by conductive ink. The oxide structures were generated in contact mode operation with a voltage in the range of −8 to −11 V applied between tip and substrate at a relative humidity (RH) of around 65% and a writing velocity of about 1 µm/s. To control the humidity of the ambient atmosphere the microscope was placed under a closed PMMA dome, which can be purged with dry nitrogen or water-vapor-saturated nitrogen. The humidity was measured by using a “SHT15” digital humidity sensor (Sensirion AG, Switzerland). Lithography of diverse patterns was performed by application of different software protocols, which were written by using a homemade software user interface. For comparative height measurements, the driving frequency, driving amplitude, setpoint and the AFM tip were kept implicitly constant, as the tip–sample interaction and therefore the measured topography depends strongly on these parameters for noncontact AFM operation.

Binding of OTS and 11-bromoundecyltrichlorosilane (both ABCR, Germany) was carried out by immersion of the structures into a 10 mM solution of the silane in toluene (spectroscopic grade, Merck, Germany). After a specific amount of time, the sample was removed from the solution and rinsed thoroughly in ultrasonic baths of toluene, dichloromethane and ethanol (all were spectroscopic grade, Merck, Germany). APTES (aminopropyltriethoxysilane, Sigma Aldrich, USA) was bound by immersion under the same conditions and preparation steps as the trichlorosilanes. For sparging of the silane/toluene solutions, argon was used as the inert gas. Binding of FITC to the APTES-functionalized structures was achieved by immersion of the samples in a low-millimolar solution of FITC in acetone (spectroscopic grade, Merck, Germany) for 90 min and subsequent cleaning in ultrasonic baths of acetone, dichloromethane and ethanol. All silanization experiments were carried out at room temperature (21 °C).

Fluorescence investigations of the samples were performed with a home-built microscope setup. The 465 nm excitation light from a pulsed laser diode (”LDH-P-C-470”, Picoquant GmbH) with adjustable repetition rate and a narrow pulse length of 75 ps, is focused on the sample by using an objective lens (100×, NA = 0.9, “EC Epiplan Neofluar” Carl Zeiss, Germany). The fluorescence light from the sample is collected with the same objective and separated from the reflected excitation light by a dichroic mirror (”z 470 RDC”, AHF Analysetechnik GmbH, Germany) and a fluorescence filter at 480 nm (Omega Optics Inc., USA). Using a beam splitter one part of the fluorescence light is focused on an avalanche photodiode (”SPCM-AQR-14”, Perkin Elmer, USA), the other part is coupled into a spectrometer (”Shamrock SR-163/SR1-GTR-600-500”, Andor Technology, UK) with a thermoelectrically cooled CCD camera (”Newton DU971N-BV”, Andor Technology, UK) as detector. The detection range of the spectrometer was adjusted to 480–655 nm. The triggering of the position controller of the piezo scan-stage as well as the read-out of the APD signal is realized by home-written software.

## References

[1] Snow E S, Campbell P M (1994). Appl Phys Lett.

[2] Dagata J A (1995). Science.

[3] Avouris P, Hertel T, Martel R (1997). Appl Phys Lett.

[4] Calleja M, García R (2000). Appl Phys Lett.

[5] Wouters D, Hoeppener S, Schubert U S (2009). Angew Chem, Int Ed.

[6] Ravoo B J (2009). J Mater Chem.

[7] Gu J, Yam C M, Li S, Cai C (2004). J Am Chem Soc.

[8] Wouters D, Schubert U S (2003). Langmuir.

[9] Sugimura H, Nanjo S, Sano H, Murase K (2009). J Phys Chem C.

[10] Mo Y, Wang Y, Pu J, Bai M (2009). Langmuir.

[11] Graaf H, Vieluf M, von Borczyskowski C (2007). Nanotechnology.

[12] Hoeppener S, Schubert U S (2005). Small.

[13] Baumgärtel T, von Borczyskowski C, Graaf H (2010). Nanotechnology.

[14] Maoz R, Cohen S R, Sagiv J (1999). Adv Mater.

[15] Haensch C, Hoeppener S, Schubert U S (2009). Nanotechnology.

[16] Fresco Z M, Fréchet J M J (2005). J Am Chem Soc.

[17] Yang M, Wouters D, Giesbers M, Schubert U S, Zuilhof H (2009). ACS Nano.

[18] Kolb H C, Finn M G, Sharpless K B (2001). Angew Chem, Int Ed.

[19] Fabre B, Herrier C (2012). RSC Adv.

[20] Sagiv J (1980). J Am Chem Soc.

[21] Howarter J A, Youngblood J P (2006). Langmuir.

[22] Ulman A (1996). Chem Rev.

[23] Fadeev A Y, McCarthy T J (2000). Langmuir.

[24] Desbief S, Patrone L, Goguenheim D, Guérin D, Vuillaume D (2011). Phys Chem Chem Phys.

[25] Ara M, Graaf H, Tada H (2002). Appl Phys Lett.

[26] Baumgärtel T, Graaf H, von Borczyskowski C, Ara M, Tada H, Borisenko V E, Gaponenko S V, Gurin V S Proceedings of International conference Nanomeeting 2011, Reviews and short notes.

[27] Ara M, Tsuji M, Tada H (2007). Surf Sci.

[28] Tada H, Ara M, Tanaka S (2002). Mater Res Soc Symp Proc.

[29] Mirji S A (2006). Surf Interface Anal.

[30] Sawoo S, Dutta P, Chakraborty A, Mukhopadhyay R, Bouloussa O, Sarkar A (2008). Chem Commun.

[31] Wang M, Liechti K M, Wang Q, White J M (2005). Langmuir.

[32] Brandriss S, Margel S (1993). Langmuir.

[33] Plueddemann E (1991). Silane Coupling Agents.

[34] Baumgärtel T, von Borczyskowski C, Graaf H (2012). Nanotechnology.

[35] Belgardt C, Graaf H, Baumgärtel T, von Borczyskowski C (2010). Phys Status Solidi C.

[36] Penzkofer A, Wiedmann J (1980). Opt Commun.

[37] Arbeloa F L, Martínez V M (2006). Chem Mater.

[38] Imhof A, Megens M, Engelberts J J, de Lang D T N, Sprik R, Vos W L (1999). J Phys Chem B.

[39] Pasternack R M, Amy S R, Chabal Y J (2008). Langmuir.

[40] Dietz C, Zerson M, Riesch C, Franke M, Magerle R (2008). Macromolecules.

[41] Dong J, Wang A, Ng K Y S, Mao G (2006). Thin Solid Films.

[42] Sugimura H, Hozumi A, Kameyama T, Takai O (2002). Surf Interface Anal.

[43] Hozumi A, Yokogawa Y, Kameyama T, Sugimura H, Hayashi K, Shirayama H, Takai O (2001). J Vac Sci Technol, A.

[44] Graaf H, Baumgärtel T, Vieluf M, von Borczyskowski C (2008). Superlattices Microstruct.

[45] Linford M R, Fenter P, Eisenberger P M, Chidsey C E D (1995). J Am Chem Soc.

